# Supplementary material for: Machine Learning Accelerates Crystallization for Structure Determination
Source: Angew Chem Int Ed Engl. 2026 May 4;65(25):e1218503. doi: 10.1002/anie.1218503 (PMC13266958; doi:10.1002/anie.1218503)

checkCIF/PLATON report

Structure factors have been supplied for datablock(s) lcz085

THIS REPORT IS FOR GUIDANCE ONLY. IF USED AS PART OF A REVIEW PROCEDURE FOR PUBLICATION, IT SHOULD NOT REPLACE THE EXPERTISE OF AN EXPERIENCED CRYSTALLOGRAPHIC REFEREE.

No syntax errors found. CIF dictionary Interpreting this report

Datablock: lcz085

Bond precision:	C-C = 0.0084 A		Wavelength=1.54184
Cell:	a=9.0182(1)	b=18.2111(1)	c=19.5504(1)
	alpha=90	beta=94.291(1)	gamma=90
Temperature:	100 K		

	Calculated	Reported
Volume	3201.79(4)	3201.79(4)
Space group	P 21/c	P 1 21/c 1
Hall group	-P 2ybc	-P 2ybc
Moiety formula	C15 H3 Ag3 F18 N6, C7 H7 Br S	C15 H3 Ag3 F18 N6, C7 H7 Br S
Sum formula	C22 H10 Ag3 Br F18 N6 S	C22 H10 Ag3 Br F18 N6 S
Mr	1135.93	1135.94
Dx,g cm-3	2.356	2.357
Z	4	4
Mu (mm-1)	17.910	17.910
F000	2152.0	2152.0
F000'	2160.42	
h,k,lmax	11,23,24	11,22,24
Nref	6884	6634
Tmin,Tmax	0.028,0.068	0.484,1.000
Tmin'	0.005	

Correction method= # Reported T Limits: Tmin=0.484 Tmax=1.000
AbsCorr = MULTI-SCAN

Data completeness= 0.964 Theta(max)= 78.375

R(reflections)= 0.0448(5913) wR2(reflections)= 0.1136(6634)

S = 1.050 Npar= 461

The following ALERTS were generated. Each ALERT has the format
test-name_ALERT_alert-type_alert-level.
Click on the hyperlinks for more details of the test.

Alert level C

```
PLAT213_ALERT_2_C Atom F00J            has ADP max/min Ratio .....        3.4 prolat
PLAT213_ALERT_2_C Atom F00K            has ADP max/min Ratio .....        3.3 prolat
PLAT213_ALERT_2_C Atom F019            has ADP max/min Ratio .....        3.8 prolat
PLAT213_ALERT_2_C Atom F01C            has ADP max/min Ratio .....        4.0 prolat
PLAT220_ALERT_2_C NonSolvent   Resd 1  F   Ueq(max)/Ueq(min) Range        5.0 Ratio
PLAT234_ALERT_4_C Large Hirshfeld Difference F017      --C01E     .      0.16 Ang.
PLAT234_ALERT_4_C Large Hirshfeld Difference F01B      --C01D     .      0.17 Ang.
PLAT342_ALERT_3_C Low Bond Precision on  C-C Bonds ...............    0.00839 Ang.
PLAT906_ALERT_3_C Large K Value in the Analysis of Variance ......      2.647 Check
PLAT911_ALERT_3_C Missing FCF Refl Between Thmin & STh/L=    0.600          5 Report
                0  2  0,   1  8  0,   5  8  8,  -9  4 13,   1  3 16,
PLAT971_ALERT_2_C Check Calcd Resid. Dens.  1.42Ang From F01C            2.10 eA-3
PLAT971_ALERT_2_C Check Calcd Resid. Dens.  0.93Ang From F01B            1.58 eA-3
PLAT977_ALERT_2_C Check Negative Difference Density on H014       .      -0.39 eA-3
```

Alert level G

```
PLAT083_ALERT_2_G SHELXL Second Parameter in WGHT  Unusually Large      25.43 Why ?
PLAT142_ALERT_4_G s.u. on b - Axis Small or Missing .............    0.00010 Ang.
PLAT143_ALERT_4_G s.u. on c - Axis Small or Missing .............    0.00010 Ang.
PLAT153_ALERT_1_G The s.u.'s on the Cell Axes   are Equal ..(Note)     0.0001 Ang.
PLAT242_ALERT_2_G Low    'MainMol' Ueq as Compared to Neighbors of       C00W Check
PLAT242_ALERT_2_G Low    'MainMol' Ueq as Compared to Neighbors of       C016 Check
PLAT242_ALERT_2_G Low    'MainMol' Ueq as Compared to Neighbors of       C01A Check
PLAT242_ALERT_2_G Low    'MainMol' Ueq as Compared to Neighbors of       C01D Check
PLAT242_ALERT_2_G Low    'MainMol' Ueq as Compared to Neighbors of       C01E Check
PLAT434_ALERT_2_G Short Inter HL..HL Contact F00D      ..F00K     .      2.84 Ang.
                                        2-x,1/2+y,3/2-z  =      2_756 Check
PLAT720_ALERT_4_G Number of Unusual/Non-Standard Labels ..........         61 Note
              Ag01    Ag02    Ag03    Br04    S005    F006    F007    F008
              F009    F00A    F00B    F00C    F00D    N00E    N00F    N00G
              N00H    N00I    F00J    F00K    N00L    F00M    C00N    C00O
              F00P    C00Q    H00Q    C00R    H00R    C00S    H00S    F00T
              C00U    C00V    C00W    C00X    H00X    F00Y    C00Z    H00Z
              C010    C011    C012    H012    C013    C014    H014    C015
              C016    F017    C018    F019    C01A    F01B    F01C    C01D
              C01E    C01F    H01A    H01B    H01C
PLAT790_ALERT_4_G Centre of Gravity not Within Unit-Cell: Resd.  #          2 Note
              C7 H7 Br S
PLAT883_ALERT_1_G Absent Datum for _atom_sites_solution_primary ..     Please Do !
PLAT912_ALERT_4_G Missing # of FCF Reflections Above STh/L=  0.600        240 Note
PLAT941_ALERT_3_G Average HKL Measurement Multiplicity ..........         2.9 Low
```

```
PLAT969_ALERT_5_G The 'Henn et al.' R-Factor-gap value ..........      3.286 Note
              Predicted wR2: Based on SigI**2  3.46 or SHELX Weight 10.81
PLAT978_ALERT_2_G Number C-C Bonds with Positive Residual Density.         0 Info
```

```
   0 ALERT level A = Most likely a serious problem - resolve or explain
   0 ALERT level B = A potentially serious problem, consider carefully
  13 ALERT level C = Check. Ensure it is not caused by an omission or oversight
  17 ALERT level G = General information/check it is not something unexpected

   2 ALERT type 1 CIF construction/syntax error, inconsistent or missing data
  16 ALERT type 2 Indicator that the structure model may be wrong or deficient
   4 ALERT type 3 Indicator that the structure quality may be low
   7 ALERT type 4 Improvement, methodology, query or suggestion
   1 ALERT type 5 Informative message, check
```

Validation response form

Please find below a validation response form (VRF) that can be filled in and pasted into your CIF.

```
# start Validation Reply Form
_vrf_PLAT213_lcz085
;
PROBLEM: Atom F00J            has ADP max/min Ratio .....        3.4 prolat
RESPONSE: ...
;
_vrf_PLAT220_lcz085
;
PROBLEM: NonSolvent   Resd 1  F   Ueq(max)/Ueq(min) Range        5.0 Ratio
RESPONSE: ...
;
_vrf_PLAT234_lcz085
;
PROBLEM: Large Hirshfeld Difference F017     --C01E     .       0.16 Ang.
RESPONSE: ...
;
_vrf_PLAT342_lcz085
;
PROBLEM: Low Bond Precision on  C-C Bonds ..............    0.00839 Ang.
RESPONSE: ...
;
_vrf_PLAT906_lcz085
;
PROBLEM: Large K Value in the Analysis of Variance ......      2.647 Check
RESPONSE: ...
;
_vrf_PLAT911_lcz085
;
PROBLEM: Missing FCF Refl Between Thmin & STh/L=    0.600          5 Report
RESPONSE: ...
;
_vrf_PLAT971_lcz085
;
PROBLEM: Check Calcd Resid. Dens.  1.42Ang From F01C            2.10 eA-3
RESPONSE: ...
;
```

```
_vrf_PLAT977_lcz085
;
PROBLEM: Check Negative Difference Density on H014      .      -0.39 eA-3
RESPONSE: ...
;
# end Validation Reply Form
```

It is advisable to attempt to resolve as many as possible of the alerts in all categories. Often the minor alerts point to easily fixed oversights, errors and omissions in your CIF or refinement strategy, so attention to these fine details can be worthwhile. It is up to the individual to critically assess their own results and, if necessary, seek expert advice.

PLATON version of 17/09/2025; check.def file version of 17/09/2025

---

duplicate check

No duplication found

Datablock lcz085 - ellipsoid plot

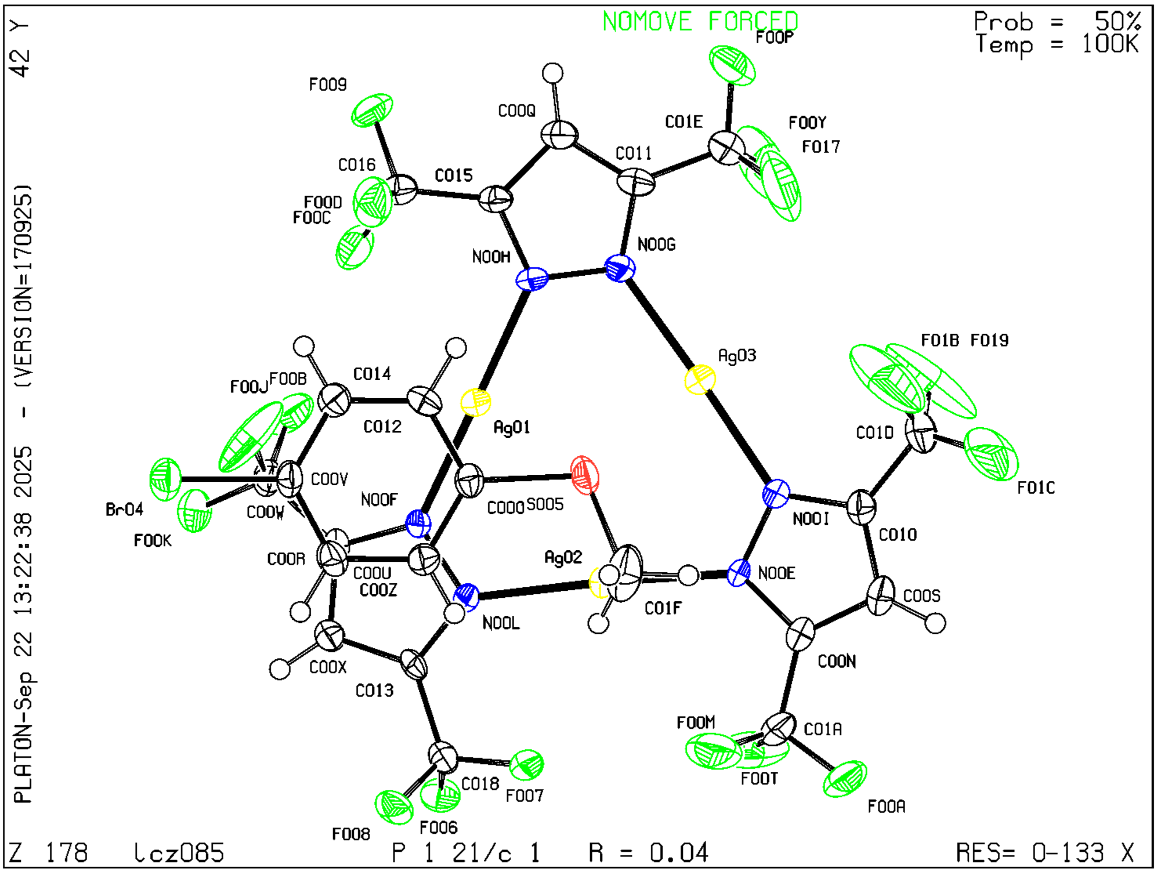

Supplement: Supplementary file 2 — Supporting File 2: anie72462‐sup‐0002‐DataFile.zip. [file ANIE-65-e1218503-s002.zip › supplementary_materials_crystallographic_data submit.zip!/cif_and_checkcif(1-114)/Ag3Pz3_85/Ag3Pz3_85_checkcif.pdf]
